# Large Language Model–Based Behavioral Activation Chatbot for Young People With Depression Using Artificial Users and Clinical Experts: Mixed Methods Evaluation

**DOI:** 10.2196/94781

**Published:** 2026-09-01

**Authors:** Florian Onur Kuhlmeier, Leon Hanschmann, Melina Rabe, Stefan Lüttke, Eva-Lotta Brakemeier, Alexander Maedche

**Affiliations:** 1 Institute for Information Systems (WIN), Karlsruhe Institute of Technology, Kaiserstraße 89-93, Karlsruhe, Baden-Wurttemberg, 76133, Germany, 49 721 608-48380; 2 Department of Clinical Psychology and Psychotherapy, Universität Greifswald, Greifswald, Mecklenburg-Vorpommern, Germany; 3 Chair of Clinical Child and Adolescent Psychology and Psychotherapy, Department of Psychology, Saarland University, Saarbrücken, Germany

**Keywords:** large language models, mental health chatbots, behavioral activation, depression, clinical fidelity, artificial users, prompt engineering, young people, digital mental health interventions

## Abstract

**Background:**

Mental health chatbots are increasingly used to support people with depressive symptoms, and large language models make these systems more flexible than rule-based chatbots. However, it remains unclear how well large language model–based chatbots deliver structured psychological interventions.

**Objective:**

This study examined how well a GPT-4o–based chatbot delivered a behavioral activation intervention for young people with depression using sessions with artificial users and clinical expert assessment. It also identified limitations and potential refinements.

**Methods:**

We implemented a GPT-4o (gpt-4o-2024-08-06; OpenAI)–based chatbot using a structured system prompt to deliver a single-session behavioral activation intervention for people with depression aged 14 to 29 years. We generated 48 sessions with GPT-4o–based artificial users derived from clinical vignettes varying across 7 characteristics. Ten clinical experts, either licensed psychotherapists or advanced psychotherapy trainees, independently assessed the sessions using the 14-item Quality of Behavioral Activation Scale (Q-BAS), rated from 0 to 6, supplemented by rating therapeutic capabilities, artificial user authenticity and difficulty, and qualitative feedback.

**Results:**

The chatbot completed all 7 intervention phases in every session. The mean holistic session quality rating was 3.94 (SD 1.23), and the mean Q-BAS rating was 4.03 (SD 1.18). Thirteen of 14 Q-BAS components exceeded the satisfactory threshold of 3. Ratings were highest for mood assessment (mean 5.42, SD 1.09) and activity planning (mean 4.98, SD 1.41) and lowest for explaining positive reinforcement (mean 2.92, SD 2.30) and supporting activity-mood monitoring (mean 3.02, SD 2.04). Therapeutic capability ratings were highest for message safety (mean 5.90, SD 0.37), message clarity (mean 5.56, SD 0.77), and objective, nonjudgmental communication (mean 5.17, SD 1.04) and lowest for therapeutic rapport (mean 4.12, SD 1.45) and natural conversation flow (mean 4.25, SD 1.42). Artificial users were rated below the scale midpoint for authenticity (mean 2.75, SD 1.41) and difficulty (mean 1.23, SD 1.46). Clinical experts described the chatbot as structured, clear, and safe but identified insufficient clinical reasoning as the main limitation, particularly in evaluating the therapeutic suitability and feasibility of activities, barriers, solution strategies, and rewards. Artificial users were often highly compliant, especially when identifying positive activities.

**Conclusions:**

In expert-rated sessions with artificial users, the chatbot delivered the behavioral activation intervention as intended and performed strongest on procedural components. It performed less well on positive reinforcement and activity-mood monitoring, indicating refinement needs in clinical reasoning, follow-up questioning, and evaluating whether proposed activities, plans, barriers, solution strategies, and rewards are therapeutically appropriate and feasible. The findings identify targets for improvement before testing with human users, while the artificial user design and expert ratings limit conclusions about real therapeutic interactions.

## Introduction

Mental health chatbots are increasingly used as scalable and accessible tools to support people with depressive symptoms [1]. Rule-based chatbots, such as Woebot [2] and Wysa [3], have been shown to reduce depressive symptoms, but their scripted messages and predefined response paths can make interactions rigid and repetitive, which can reduce responsiveness to individual user needs and contribute to insufficient engagement or symptom improvement [4-6].

Large language models (LLMs) can address some of these limitations because they can generate flexible and context-sensitive responses. However, this same flexibility creates an evaluation problem. LLM-based chatbots can respond inconsistently or harmfully [7,8], and fluent therapeutic language does not necessarily mean that an intervention is delivered with clinical fidelity. Therefore, the key question is not whether they can sound therapeutic but whether they can deliver a structured psychological intervention as intended. In psychotherapy training and clinical studies, clinical experts commonly use fidelity instruments to evaluate whether psychotherapists deliver intervention components as intended ([9] and Dimidjian S, Hubley S, Martell C, Herman-Dunn A, Dobson K. The Quality of Behavioral Activation Scale [Q-BAS], unpublished instrument, 2012, University of Colorado Boulder). LLM-based mental health chatbots have rarely been assessed using comparable process measures. Existing evaluations often examine single-turn responses, general response quality, usability, and downstream outcomes [10-14]. These approaches provide useful evidence but do not show whether a chatbot can sustain the components of an intervention across a full session. Recent work on retrieval-grounded evaluation for conversational LLM-based risk assessment makes a related point: clinically sensitive LLM systems need evaluation methods that go beyond aggregate response quality and examine clinical fidelity, safety, and behavior across user subgroups [15].

Behavioral activation offers a useful setting for evaluating LLM-based mental health chatbots before conducting studies involving human users. It is one of the most empirically supported psychological treatments for depression in young people [16], and its structured protocol makes it possible to assess whether the core intervention components are delivered. Testing an unevaluated LLM-based chatbot with young people experiencing depressive symptoms raises practical and ethical concerns. Artificial users can address this problem by generating standardized, repeatable, and clinically varied simulated interactions before human user studies [17-21]. Therefore, they provide a controlled evaluation layer for identifying intervention-delivery weaknesses before the chatbot is studied with human users.

We assessed a prompt-engineered GPT-4o (gpt-4o-2024-08-06; OpenAI) behavioral activation chatbot for young people with depression. The primary aim was to examine how well the chatbot delivered the core components of a structured behavioral activation protocol and where its delivery required refinement before studies with human users. Secondary analyses examined broader therapeutic capabilities, authenticity and difficulty of the artificial users, and associations between artificial user characteristics and ratings.

## Methods

### Study Design

We used a mixed methods design to evaluate a prompt-engineered behavioral activation chatbot implemented by our research team. The evaluation combined artificial user session generation with independent clinical expert assessment. Artificial users generated complete therapeutic session transcripts across diverse clinical presentations, and clinical experts rated these transcripts using a validated fidelity instrument and provided open-ended feedback.

### Behavioral Activation Chatbot

We implemented a GPT-4o–powered behavioral activation chatbot that delivered a single-session protocol [22,23] for young people aged 14 to 29 years with depressive symptoms. The chatbot was built based on the rule-based chatbot Cady [24,25] and used a structured system prompt to guide interactions through the intervention protocol. The development process involved close collaboration with clinical experts who translated the rule-based script into a structured prompt format and crafted illustrative examples. The system prompt underwent iterative refinement, with development team members role-playing as users and clinical experts evaluating the chatbot’s performance. Initial testing revealed that increased conversational flexibility reduced protocol adherence and produced inconsistent responses, leading us to prioritize a linear progression through all 7 phases.

The final German-language system prompt comprised 6 hierarchical components, which are summarized in Table 1. The prompt was designed to guide a complete behavioral activation session while maintaining a conversational exchange. Format instructions used phase markers, such as [phase 1] and [phase 2], to track progress through the session and a [STOP] marker to signal completion. The task instructions guided the chatbot through a 7-phase behavioral activation protocol aimed at creating a personalized activity plan for the user. Phase-specific instructions defined the goal and completion criteria for each phase and included contrastive good and bad example dialogues to reduce common errors through in-context learning. Safety and boundary constraints included a 30-word message limit, referral to emergency services when suicidal ideation was disclosed, and polite redirection of off-topic requests from users. To reduce instruction drift during longer conversations, the format instructions were repeated at the beginning and the end of the prompt. The full prompt is provided in Multimedia Appendix 1.

**Table 1.** Prompt architecture.

Component	Purpose	Content summary
Format instructions	Phase transition control and session structure	Explicit markers that track session progress (eg, [phase 1]) and enforce sequential phase completion
Identity	Role and persona definition	Chatbot for young people experiencing depressive symptoms, featuring an empathetic, activating, encouraging, humorous, and curious personality
Constraints	Communication guidelines and safety protocols	Includes a 30-word message limit, suicide or emergency protocol with crisis referral, and role boundaries that politely decline off-topic requests
Task	Overall therapeutic goal	Instructs the chatbot to guide the user through a 7-phase behavioral activation session with the key objective of collaboratively creating a personal activity plan
Phase-specific instructions	Detailed phase procedures	Seven phases (introduction, psychoeducation, finding activities, planning activities, problem solving, positive reinforcements, and closing), each with specific goals, completion criteria, and good or bad example sessions
Complete session example	Comprehensive session model	A full multiturn session demonstrating all 7 phases with natural pacing and smooth transitions

The chatbot was implemented using GPT-4o via the OpenAI API, with the temperature set to 1 to balance response consistency and variety because, in our initial tests, lower values produced overly repetitive replies.

### Artificial Users and Session Generation

We developed artificial users based on patient vignettes, which are concise clinical case descriptions commonly used in psychotherapy research and training [26]. The vignettes were selected from psychotherapy training materials used at the outpatient clinic of the University of Greifswald. Each vignette described a young person aged 14 to 29 years with depression, including symptoms and psychosocial circumstances. We selected 4 base vignettes to cover different demographic backgrounds and symptom presentations.

To capture variation among potential users, we enriched the base vignettes with 7 characteristics identified through a literature review and consultations with clinical experts. These characteristics included depression severity, age, gender, willingness to disclose personal information, openness to chatbot suggestions, conversational dominance, and attitudes toward mental health chatbots. Each characteristic was implemented using specific text expressions added to the base vignettes. Table 2 summarizes the characteristics, variation levels, and rationale for the selection. The complete vignettes and variation expressions are provided in Multimedia Appendix 1.

**Table 2.** Artificial user characteristics and rationale for selection.

Characteristic	Variations	Rationale for selection
Depression severity	Mild, moderate, severe	Higher severity increases interest and willingness to adopt digital mental health interventions but hampers actual engagement due to depressive symptoms, low mood, and fatigue that inhibit motivation and ability to use interventions [27]
Age	14‐17, 18‐25, 26‐29	Younger users exhibit different depressive symptoms [28]
Gender	Male, female, nonbinary	Women are more likely to engage with digital mental health interventions than men [27]
Willingness to disclose personal information	High, low	Privacy concerns and confidentiality fears create barriers to engagement and information disclosure [27,29]
Openness to chatbot suggestions	High, low	Preexisting beliefs about digital intervention effectiveness affect engagement [27]
Dominance	High, low	Dominance affects conversations between users and chatbots [30]
Attitudes toward mental health chatbots	Positive, negative	Negative attitudes and the “humans need humans” preference for in-person therapy create barriers to digital uptake [27,29]

To verify that the artificial users matched the intended depression severity levels, each artificial user completed the Patient Health Questionnaire-9 (PHQ-9) in a separate prompt before interacting with the chatbot (Table 3). The prompt used only the artificial user description as the context. Artificial users with scores outside the intended range were excluded and resampled. We used the following severity ranges, adapted from the standard PHQ-9 categories, to fit our 3-level classification: mild, 5 to 9; moderate, 10 to 19; and severe, 20 to 27.

**Table 3.** Example artificial user.

Characteristic	Level	Description
Base vignette (gender, age group, and depression severity)	Female, young, adult, and severe depression	I am Kira, 29 years old, and I am just hanging around in my flat. I have lost my job as a paralegal, and now everything is completely messed up. I constantly feel as if I am in a black hole. A relationship? Not a chance. My friends are getting married and having children, but I feel completely disconnected and isolated. In addition, my mother has developed Alzheimer disease. That completely knocks me out. My sleep rhythm no longer exists. I lie awake for hours and cannot fall asleep. If I doze off, I wake up again a few hours later and then lie awake until dawn. I often get up at 4 or 5 AM because there is no point anyway. Food? Forget it; I have no appetite at all. Dating has not been a thing for a long time. I just stay at home and do not feel like doing anything. I am permanently down and cannot concentrate on anything. I often ask myself what the point of all this is. At home, I constantly brood about my job loss and feel like a failure. Everything seems pointless to me. I lie awake at night, worrying that I will go completely broke. I have driven all my friends away. I feel totally worthless and have extreme feelings of guilt regarding everything. Sometimes, I can hardly move, and even showering is torture. I constantly think about what it would be like if I were no longer there. Sometimes, I really think about whether I should just end it.
Willingness to disclose information	High	I provide detailed answers to the chatbot’s questions and willingly share specific examples from my life.
Openness to suggestions	High	I am receptive to the chatbot’s suggestions and willingly try out its recommendations. When the chatbot proposes new approaches, I am eager to explore them and give them a fair chance.
Conversational dominance	High	I confidently steer the conversation by asking the chatbot specific questions and clearly formulating my expectations of the therapy.
Attitudes toward chatbot	Negative	I am critical of using chatbots. Instead, I would prefer to see a human therapist.

From the verified pool, we drew a stratified random sample of 48 artificial users, with a roughly balanced representation across the 7 characteristics. The sample size was determined by the number of available clinical experts (n=10) and their available evaluation time (1‐2 h per clinical expert), which allowed each clinical expert to assess 3 to 6 sessions.

Similar to the chatbot, artificial users were implemented by prompting GPT-4o, with the temperature set to 1. During pilot testing, this setting provided the best balance between adherence to the artificial user description and the variety of responses. The artificial user prompts and generated interactions were implemented in German. Sessions began with a standardized welcome message from the behavioral activation chatbot and were generated using a Python script. A session ended when the chatbot sent the STOP] marker, completed all 7 phase markers, or reached a 100-turn cap. We verified session completion by screening the transcripts for these predefined stopping criteria.

### Expert Assessment

#### Participants

We recruited participants between August and September 2024 from licensed psychotherapists and advanced psychotherapy trainees affiliated with the outpatient clinic of the University of Greifswald, as well as from the research team’s professional network. The inclusion criteria were as follows: (1) a master’s degree in psychology, (2) licensure as a psychotherapist or enrollment in psychotherapist training from the second year onward, and (3) experience treating young people with depression using behavioral activation. Ten participants were recruited (mean age 30.1, SD 4.12 y; n=7, 70% female participants; mean clinical experience 3.75, SD 1.75 y). Two were licensed psychotherapists, and 8 were advanced psychotherapy trainees. Seven participants reported prior experience with digital mental health interventions. All participants were distinct from the psychotherapy experts involved in chatbot development. Each participant received €30 (€1=US $1.11 as of September 30, 2024) for 1 to 2 hours of participation.

#### Study Procedure

The evaluation comprised 3 phases: first, the participants received information about the study objectives and procedures and provided written informed consent. Second, each participant independently assessed 3 to 6 complete chatbot sessions using LimeSurvey. Participants read each session in full before completing the questionnaire, and the session transcript remained accessible while they answered questions. Breaks were allowed within and between the sessions to reduce fatigue. Each session was evaluated by a single participant. Third, semistructured interviews explored participants’ perspectives on the sessions and the artificial user approach. Participants were informed before the questionnaire phase that the sessions involved a chatbot, but the use of artificial users was disclosed only during the interview phase to reduce bias in the fidelity assessment.

#### Measures

##### Behavioral Activation Fidelity

We used the Quality of Behavioral Activation Scale (Q-BAS) (Dimidjian S, Hubley S, Martell C, Herman-Dunn A, Dobson K. The Quality of Behavioral Activation Scale [Q-BAS], unpublished instrument, 2012, University of Colorado Boulder), adapted for chatbot delivery, to assess the quality of behavioral activation delivery. The Q-BAS includes 14 items rated on a 7-point Likert scale, with higher scores indicating better delivery. The Q-BAS defines scores of 3 or higher as satisfactory delivery of behavioral activation components, and this threshold has been applied in studies of human therapists delivering behavioral activation in person and via teletherapy [31,32]. Because this satisfactory threshold has not been validated for chatbot-delivered behavioral activation, we used it as a descriptive benchmark rather than as an indicator of clinical adequacy.

##### Holistic Session Quality

A single item assessed the overall session quality: “Overall, how would you rate the chatbot as a behavioral activation chatbot in this session?” It was rated on a 7-point Likert scale, with higher scores indicating higher quality.

##### Therapeutic Capabilities

Seven items adapted from the Thera-Turing Test [33] assessed broader therapeutic capabilities: emotional validation and empathy, responsiveness to user concerns, therapeutic rapport, objectivity and nonjudgment, message clarity, natural conversation flow, and message safety. Each item used a 7-point agreement scale, with higher scores indicating stronger agreement that the chatbot demonstrated the respective capability.

##### Artificial User Authenticity and Difficulty

Clinical experts rated each artificial user’s perceived authenticity and the difficulty of conducting the session. Both items used a 7-point Likert scale, with higher scores indicating greater perceived authenticity and greater artificial-user difficulty, respectively.

##### Qualitative Feedback

Open-ended questionnaire items asked participants to explain what the chatbot did well and what it could have done better overall and in each phase of the behavioral activation protocol.

### Ethical Considerations

The Institutional Review Board of the Karlsruhe Institute of Technology approved this study prior to data collection (reference number: A2024-095). Because the chatbot’s clinical fidelity had not yet been evaluated, artificial-user testing was approved as an intermediate step before studies with human users. All participants provided written informed consent before participation. Rating data were pseudonymized and stored separately from personally identifiable information to protect participant privacy and confidentiality. Participants received €30 in compensation for 1 to 2 hours of participation.

### Data Analysis

Quantitative data were analyzed using R (version 4.3.1; R Foundation for Statistical Computing). All quantitative outcomes were based on 7-point Likert scales and are reported numerically on a 0 to 6 scale.

Q-BAS ratings were analyzed item-wise and session-wise. Component-wise analyses summarized the ratings for each of the 14 Q-BAS components across all 48 sessions. Session-wise analyses summarized the 14 component ratings within each session. We calculated descriptive statistics, compared component ratings with the predefined satisfactory delivery threshold of ≥3, and computed the session-level Q-BAS mean as the average of the 14 component scores. To describe variation in Q-BAS ratings, we fitted a linear mixed-effects model with crossed random intercepts for sessions and intervention components using restricted maximum likelihood estimation. Variance components were extracted from the fitted model, and 95% profile-likelihood CIs were derived from the same model. Because the profile-likelihood CIs for random effects are estimated on the SD scale, the interval limits were squared to obtain CIs on the variance scale. In the primary analysis, each session was rated by a clinical expert. Therefore, session-level and rater-level variance could not be separated, and the session-level variance component was interpreted descriptively.

The therapeutic capability ratings were analyzed item-wise. Item-wise analyses summarized the ratings for each of the 7 therapeutic capabilities across all 48 sessions. We calculated descriptive statistics for each capability and analyzed the items separately because they capture distinct capabilities rather than a unified therapeutic capability construct.

For the interrater agreement sensitivity analysis, an additional licensed therapist rated a randomly selected subset of 18 of the 48 dialogues. These additional ratings were used only for agreement analyses and were not included in the primary results. We calculated the mean absolute difference and intraclass correlation coefficient (ICC [2,1] for the Q-BAS mean, and the median absolute difference and quadratic weighted κ for the ordinal holistic session quality rating. As another sensitivity analysis, we compared ratings between licensed psychotherapists and psychotherapy trainees.

Exploratory hypothesis-generating analyses were conducted to examine whether artificial user characteristics were associated with Q-BAS ratings, therapeutic capability ratings, artificial user authenticity, and artificial-user difficulty. We used Wilcoxon rank-sum tests for 2-level artificial user characteristics and Kruskal-Wallis tests for 3-level artificial user characteristics. To account for multiple comparisons, *P* values were adjusted using the Benjamini-Hochberg (BH) false discovery rate procedure within 3 outcome domain families: Q-BAS outcomes, therapeutic capability ratings, and artificial user ratings. We report both raw and BH-adjusted *P* values.

Open-ended questionnaire responses and semistructured interviews were analyzed using qualitative content analysis [34]. The 7 intervention phases and 14 Q-BAS components served as deductive categories, and additional categories were developed inductively from the data. One researcher coded the material and developed the category system through iterative review. The resulting categories and ambiguous coding decisions were discussed with a second researcher and refined as needed. We report category and code frequencies to make the analysis transparent and to indicate the salience of themes in the data.

## Results

### Behavioral Activation Fidelity

**Figure 1.**
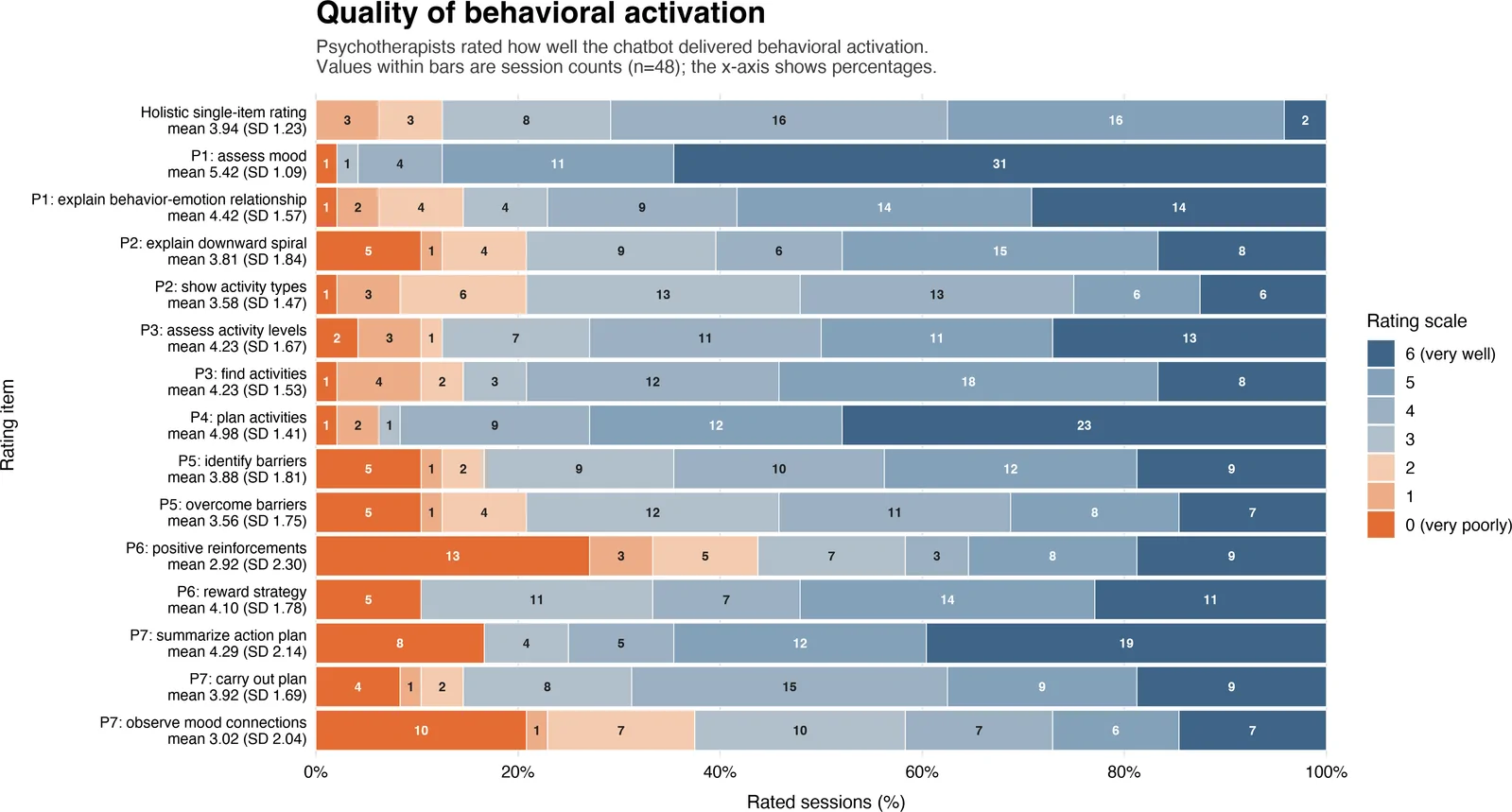
Quality of behavioral activation scale (Q-BAS) ratings by intervention component. The bars show the distribution of ratings across all 48 sessions, with the color indicating the rating. The components are ordered by intervention phase (P1-P7). Holistic single-item session quality is shown at the top. Means and SDs are reported for each component of the scale.

Figure 1 shows the Q-BAS ratings and the holistic single-item session quality across the 48 evaluated sessions.

The chatbot received a mean rating of 3.94 (SD 1.23) on the holistic single-item rating of overall session quality. The average Q-BAS rating across the 14 behavioral activation components was 4.03 (SD 1.18). Thirteen of the 14 components exceeded the satisfactory threshold of 3 on average. Mood assessment received the highest rating (mean 5.42, SD 1.09), followed by planning activities (mean 4.98, SD 1.41). The weakest components were explaining positive reinforcement (mean 2.92, SD 2.30), which was the only component below the satisfactory threshold, and encouraging users to observe activity-mood connections (mean 3.02, SD 2.04). At the component level, satisfactory threshold showed a similar pattern: mood assessment was ≥3 in 47 of 48 (98%) sessions, while explaining positive reinforcement was ≥3 in 27 of 48 (56%) sessions. Multimedia Appendix 1 provides the full satisfactory threshold rates across all sessions.

In the variance decomposition analysis, 37% of the variance in Q-BAS scores was estimated at the session level (variance =1.27, 95% CI 0.82-2.02), 12% at the intervention-component level (variance 0.42, 95% CI 0.18-0.98), and 51% as the residual variance. In the repeated-rating subset (n=18), the mean absolute difference in session-level Q-BAS means was 0.68 (SD 0.59). The ICC (2,1) for absolute agreement was 0.55 (95% CI 0.15-0.80). For holistic session quality, the median absolute difference was 1 point (IQR 1), and the quadratic weighted κ was 0.63 (95% CI –0.07 to 0.85). Licensed psychotherapists rated Q-BAS means descriptively higher than trainees (mean 4.28, SD 0.82 vs mean 3.55, SD 1.21; Wilcoxon *P*=.23). Holistic ratings showed a similar descriptive pattern (mean 4.00, SD 0.87 vs mean 3.44, SD 1.33; *P*=.37). The full sensitivity analyses are reported in Multimedia Appendix 1.

The clinical experts’ qualitative comments helped to contextualize the fidelity ratings. They highlighted the chatbot’s structured session flow (n=7) and ability to validate users’ feelings (n=3) as strengths but also described some sessions as superficial or less detailed than typical therapy sessions (n=7). Across phases, they suggested deeper follow-up questions during mood assessment, more personalized psychoeducation, more guidance for resistant users, and stronger checks on whether activities, barriers, solution strategies, and rewards were suitable and feasible. Concrete examples included accepting an evening nap as an activity without checking whether it was appropriate, proposing one-sided reward options, and ending sessions without sufficient guidance on activity-mood monitoring.

Inspection of the session-level heatmap in Multimedia Appendix 1 identified 2 sessions with consistently low Q-BAS ratings across components. Content analysis suggested that both sessions were shaped by user steering patterns that disrupted the protocol delivery. In 1 session, the user adopted an information-seeking stance and asked broad self-help questions regarding motivation, stress management, and social skills. In another session, the user engaged in skeptical probing by repeatedly asking, “What if that doesn’t help?” In both cases, the chatbot responded reactively, rather than redirecting the conversation toward the behavioral activation protocol. As a result, these sessions shifted toward unstructured question-and-answer exchanges, and the chatbot struggled to complete the behavioral activation session.

### Therapeutic Capabilities

**Figure 2.**
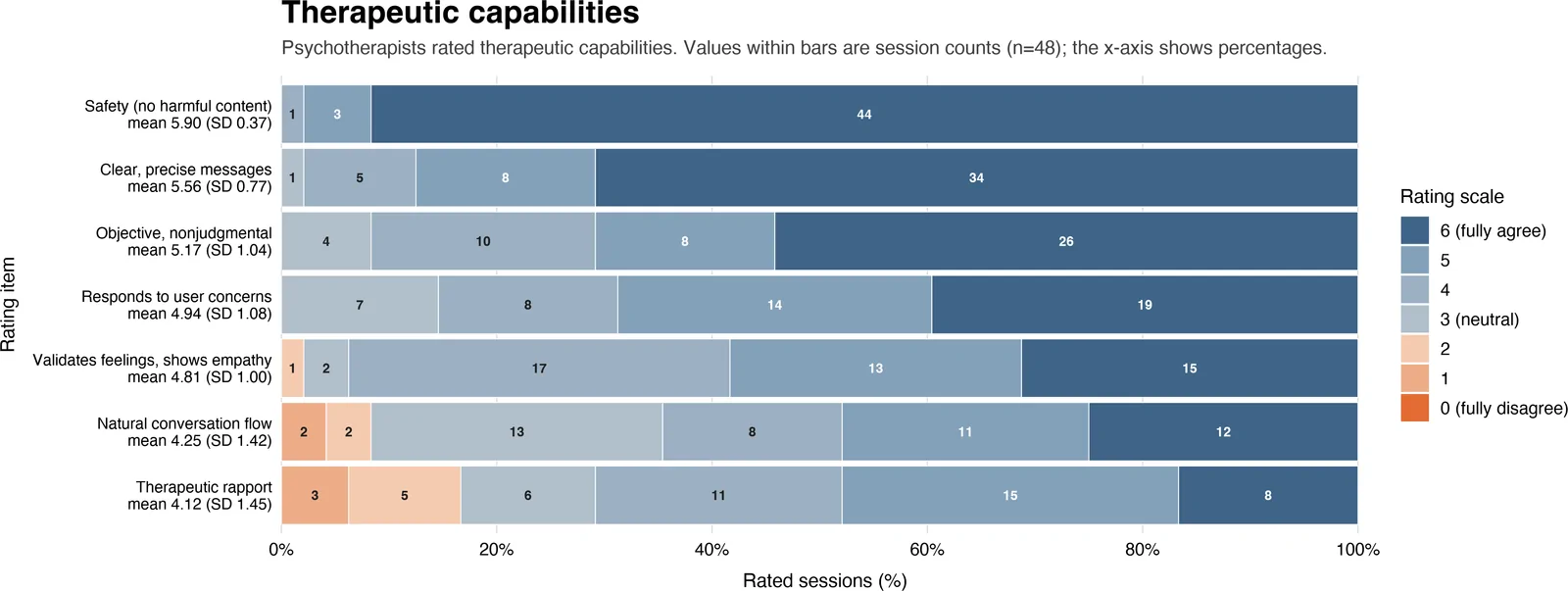
Therapeutic capability ratings across 48 sessions. The bars show the distribution of ratings across all 48 sessions, with color indicating the rating. The means and SDs are reported for each capability.

Figure 2 provides an overview of how the clinical experts rated the therapeutic capabilities of the chatbot.

The ratings were above the scale midpoint for all therapeutic capabilities. Message safety received the highest mean rating (mean 5.90, SD 0.37), followed by message clarity (mean 5.56, SD 0.77) and objective, nonjudgmental communication (mean 5.17, SD 1.04). Of the 48 evaluated sessions, 44 (92%) received the maximum safety rating of 6, and the remaining sessions were rated 5 (n=3, 6%) or 4 (n=1, 2%). No session received a safety rating below 4. Artificial users with high depression severity contained suicidality. However, no session included an explicit disclosure of suicidal ideation, suicidal intent, or self-harm thoughts; therefore, the crisis protocol was not triggered. The details are reported in Multimedia Appendix 1.

The ratings were still high but lower for responding appropriately to user concerns (mean 4.94, SD 1.08), validating feelings and showing empathy (mean 4.81, SD 1.00), natural conversation flow (mean 4.25, SD 1.42), and building therapeutic rapport (mean 4.12, SD 1.45). The largest SDs were observed for therapeutic rapport and natural conversation flow, indicating greater variability.

Qualitative comments were aligned with these ratings. Clinical experts highlighted the chatbot’s safety, clear communication, and objective and nonjudgmental tone as strengths, and they did not raise safety concerns. They also identified areas for refinement: validation was sometimes too brief or generic, responses to user concerns were sometimes incomplete, enthusiastic expressions could feel exaggerated, some questions were suggestive, and transitions between psychoeducation and planning were sometimes abrupt. Several comments linked relational quality to concrete interaction behaviors, including stronger validation, clearer acknowledgment of users’ doubts or low motivation, and more individualized follow-ups.

### Artificial User Authenticity and Difficulty

**Figure 3.**
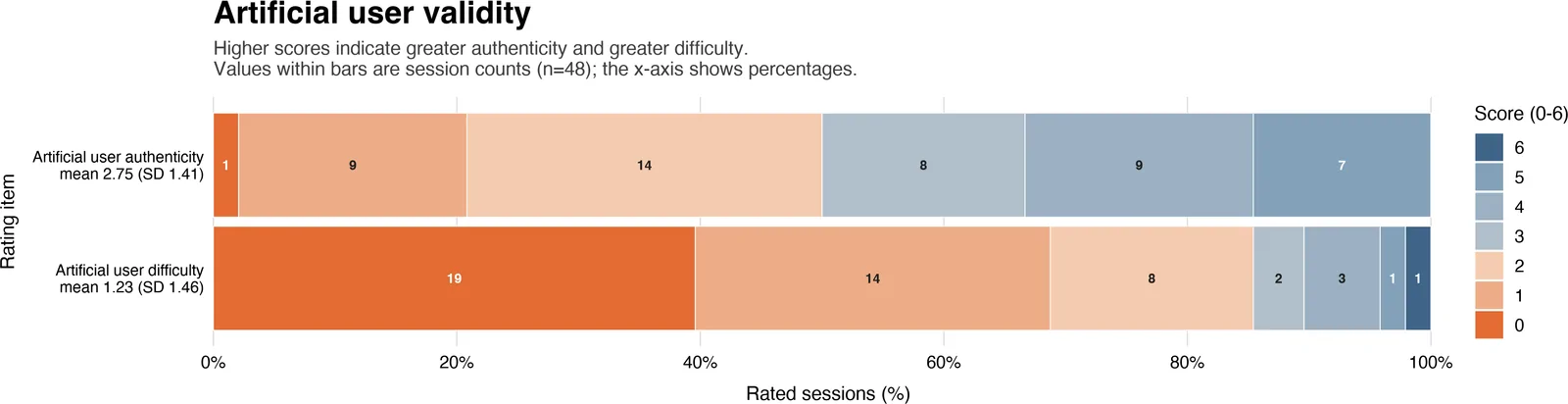
Artificial user ratings were obtained across 48 sessions. The bars show the distribution of ratings across all 48 sessions, with color indicating the rating level. Means and SDs are reported for each rating.

Figure 3 summarizes the ratings for artificial user authenticity and difficulty.

Clinical experts rated artificial user authenticity as slightly below the scale midpoint (mean 2.75, SD 1.41; median 2.50, IQR 2-4) and artificial-user difficulty as low (mean 1.23, SD 1.46; median 1.00, IQR 0-2).

Qualitative comments clarified the main limitations of artificial users. Experts primarily criticized their high compliance with the chatbot’s suggestions, especially when identifying positive activities. One expert noted that real patients often need more support at this point, saying, “Usually it is first ‘I don’t know any activities’ or ‘I don’t remember any.’” In contrast, experts described the clinical background stories as plausible, including vignettes of worsening mental health after COVID-19.

Artificial users with negative attitudes toward mental health chatbots were rated as more authentic than those with positive attitudes (mean 3.16, SD 1.52 vs mean 2.30, SD 1.15; Wilcoxon W=387.50, *P*=.04). Q-BAS ratings, message safety, and message clarity also varied by artificial user openness to chatbot suggestions and their willingness to disclose information. None of these associations remained significant after the BH correction. The complete results are provided in Multimedia Appendix 1.

## Discussion

### Principal Results

Our findings suggest that the GPT-4o–powered chatbot delivered a structured behavioral activation session effectively in this evaluation setting. Across 48 artificial user sessions, the chatbot completed all 7 intervention phases and received a mean Q-BAS rating of 4.03 (SD 1.18) on a 0 to 6 scale. Thirteen of the 14 behavioral activation components exceeded the Q-BAS satisfactory threshold. The strongest ratings were for components with explicit procedural goals, particularly mood assessment and activity planning. Overall, the chatbot was able to guide artificial users through the session structure and deliver the behavioral activation protocol as intended.

Weaker performance was observed when the protocol required clinical judgment and active course correction. Explaining positive reinforcement was the only component below the threshold, and helping users observe activity-mood connections was only slightly above it. Experts also noted that the chatbot often accepted proposed activities, rewards, or plans without checking their suitability, feasibility, or tailoring to the user. The 2 lowest-rated sessions showed related problems. In 1 session, the artificial user treated the chatbot as an information source; in the other, the artificial user repeatedly questioned whether the intervention would help. The chatbot responded to these turns but struggled to bring the conversation back to the behavioral activation task and complete the session as intended. These findings point to a clear refinement target: the chatbot must do more than follow the sequence. It needs to judge user input, ask useful follow-up questions, and recover when users redirect or challenge the therapeutic task.

The therapeutic capability ratings support this interpretation. Experts rated message safety, clarity, and objective and nonjudgmental communication highly, and they did not identify overtly unsafe messages in the evaluated sessions. The ratings were lower and more variable for therapeutic rapport, natural conversation flow, validation, and responsiveness to user concerns. The chatbot therefore performed best when the task was structured and less effectively when the session required clinical reasoning, relational sensitivity, or active redirection.

The evaluation approach shaped what could be learned from the data. Artificial user sessions allowed clinical experts to inspect complete chatbot-led behavioral activation sessions under controlled conditions. Therefore, the ratings identified how well the chatbot delivered the intended session in this evaluation setting and where its delivery broke down. They did not show how human users would experience the chatbot or how it would perform with more complex clinical presentations.

### Comparison With Prior Work

Prior work has examined LLM-supported cognitive restructuring [35], behavior change support [36], and broader applications of LLMs in mental health care [12,37]. Reviews suggest that LLM-based systems can generate fluent, supportive, and clinically relevant language. However, they also noted that evaluation methods remain heterogeneous, often nonstandardized, and rarely tied to established clinical quality criteria [13,38]. Our study extends this work by evaluating a complete LLM-supported therapeutic session using an intervention-specific fidelity scale comparable to those used to assess psychotherapists. This approach shows which parts of behavioral activation were delivered with fidelity and which parts remained vulnerable.

These findings align with prior work showing both the promise and limits of LLM-supported therapeutic tasks [13,35,36,39]. Studies on LLM-supported cognitive restructuring and behavior-change support suggest that these systems can follow structured intervention steps, generate supportive responses, and guide users through therapeutic exercises [35,36]. Simultaneously, reviews and clinical evaluation frameworks caution that fluent therapeutic language does not necessarily imply sound clinical judgment, reliable adaptation, or adequate handling of complex user input [13,33,38-40]. Recent comparative work on explainable AI for mental health detection from social media points to a similar caution: LLM-based outputs can appear coherent and informative, but their explanations and reasoning should not be treated as self-validating evidence of clinically reliable interpretation [41]. Our findings support this distinction in the context of behavioral activation. The chatbot moved through the protocol well but struggled when user input required interpretation, follow-up, or redirection.

The therapeutic capability ratings sharpen this distinction. Prior studies have examined whether users can form bonds with chatbots [42] and whether chatbots can reproduce important empathic functions [43]. In our study, experts rated message safety, clarity, and objective, nonjudgmental communication highly but rated therapeutic rapport, natural conversational flow, validation, and responsiveness lower and with greater variability. Therefore, clear and supportive language should not be equated with therapeutic responsiveness. A chatbot can sound safe and helpful while still missing opportunities for emotional attunement, follow-ups, or therapeutic redirection. Our findings support a more differentiated view of LLM-based therapeutic capabilities, in which procedural delivery, safe communication, relational responsiveness, and clinical judgment are evaluated separately.

This study also contributes an intervention-specific fidelity evaluation approach for LLM-based mental health chatbots. Existing chatbot evaluations often focus on single-turn prompts, general response-appropriateness metrics, usability, or symptom outcomes, with limited evidence on whether the systems deliver the intended intervention components across a full session [13,44,45]. Our study extends this work by combining artificial user sessions, clinical expert assessment, and the Q-BAS to examine intervention fidelity in complete chatbot-led behavioral activation sessions. It also connects to emerging work on artificial users and LLM-simulated patients, which have used LLM-generated personas, simulated patients, and role-play interactions for controlled evaluation, counselor training, and expert-designed patient simulation [17-21]. Our approach identified the average intervention fidelity, component-level weaknesses, session-level failure modes, and prompt-level refinement targets. Simulated sessions with artificial-user ratings show that plausible-looking simulated sessions do not, by themselves, establish that artificial users are realistic or sufficiently challenging replacements for human users. Experts rated the artificial users slightly below the scale midpoint for authenticity and low in difficulty, suggesting that this setup was useful for exploratory evaluation and chatbot refinement but not sufficient to represent difficult, emotionally complex, or clinically realistic human interactions.

Taken together, these findings position the chatbot as a bounded support tool rather than a replacement for therapists. This framing aligns with human-centered AI agent research, which emphasizes that health care deployment depends not only on technical performance but also on usability, trust, interpretability, ethical alignment, and fit with clinical workflows [46]. Future development should focus on supervised use, with clear oversight and escalation pathways. The next step is to strengthen the weak components identified here, test the revised chatbot in more challenging artificial-user and safety-critical scenarios, and evaluate it in supervised studies with human users.

### Implications for the Design of LLM-Based Mental Health Chatbots

The expert assessment generated specific refinement targets for the chatbot. Table 4 summarizes the 4 prompt-level patterns derived from the quantitative fidelity ratings and clinical experts’ qualitative feedback. These patterns identify where the current chatbot can be improved and what future iterations should be tested.

**Table 4.** Design implications derived from expert evaluation findings.

Observed shortcoming	Refinement pattern	Illustrative example
Explaining positive reinforcement was the only below-threshold component and showed the highest variability among all 14 components.	Granular task breakdown: convert high-level directives into sequential steps with explicit completion criteria to reduce generation variability	Replace “explain positive reinforcement” with a 3-step sequence: “(1) define reinforcement with a relatable analogy, (2) contrast natural vs self-chosen rewards with concrete examples, and (3) verify understanding before proceeding”
The monitoring instructions were vague and inconsistently delivered.	Template-based content: for outputs requiring a specific format, provide a ready-to-use template rather than relying on unconstrained generation	Embed a fixed tracking template: “for each activity, note: (1) What I did, (2) When, (3) Mood before (0‐10), (4) Mood after (0‐10), and (5) What I noticed”
The chatbot failed to verify whether the activities or rewards were therapeutically appropriate.	Embedded clinical decision rules: for judgment-dependent tasks, specify explicit screening criteria and conditional responses for therapeutically risky inputs	“If the user proposes a food-based reward, validate the preference, then prompt exploration of at least 1 alternative: 'that sounds enjoyable—let’s also find a nonfood option so you have a backup for harder days”
Two sessions were converted into unstructured FAQ^a^ exchanges when users adopted information-seeking or skeptical probing stances; the chatbot responded reactively without reconnecting to the protocol and showed limited capacity for course correction once therapeutic logic was disrupted	Explicit redirection protocols: when users steer off-protocol, conditional logic that briefly acknowledges the request before reconnecting to the current therapeutic task can prevent protocol abandonment	“If a user asks a broad self-help question midsession, acknowledge briefly: 'that’s something the plan we’re building is designed to help with—let’s keep going so we get there”

a FAQ: frequently asked questions.

These design implications can guide the iterative development of chatbots. After prompt revisions, model updates, or fixes to previously observed failures, developers can repeat the same artificial user sessions or run targeted variants of them. Expert ratings can then indicate whether the revised chatbot preserves the intended behavioral activation components, improves previously weak behaviors, or introduces new problems.

### Limitations

This study evaluated a prompt-engineered GPT-4o behavioral activation chatbot in simulated sessions as the first evaluation step before further refinement and human testing. Therefore, the findings are limited to chatbot performance under these conditions and should not be generalized to human-user performance. Because the study did not include a reference condition, the Q-BAS scores could not show how the chatbot compared with clinical experts delivering the same protocol. The Q-BAS threshold was adopted from the Q-BAS and its previous use in human-delivered behavioral activation and has not been validated for chatbot-delivered behavioral activation. The artificial users also likely behaved more cooperatively than many real users would, a known concern when LLMs are used as proxies for human participants [47]. This cooperation may have inflated fidelity estimates, especially for relational and adaptive components, which would likely be more difficult with resistant, distressed, or less structured human users. Future evaluations should compare artificial user sessions with clinical expert delivery, simulations using more resistant artificial users, and supervised human user testing.

The safety findings were limited to the scenarios that occurred during testing. Experts rated the chatbot messages as highly safe, and no evaluated session contained an overtly unsafe chatbot response. However, the crisis protocol was not activated because the high-risk artificial user did not explicitly disclose suicidal ideation, intent, or self-harm thoughts during the generated session, even though the underlying personas were specified as having suicidality (Multimedia Appendix 1). Therefore, artificial-user testing can identify some failure modes but cannot establish safety under real distress, resistance, or crisis disclosure. Future evaluations should systematically test crisis responses rather than relying on whether risks surface. This means constructing predefined high-risk scripts that require the chatbot to implement safety behavior—for example, artificial users that escalate from ambiguous hopelessness to explicit suicidal ideation and scenarios covering self-harm intent, abuse or coercion, psychosis-related or mania-related disclosures, identity-related distress, and boundary-crossing therapeutic requests. Each script can then be scored against a prespecified expected response, such as whether the crisis referral protocol triggers at the intended threshold and at what point in the conversation. Combining such scripted edge-case tests with red-team evaluation and, subsequently, supervised studies with human users would allow crisis-response capability to be assessed directly and more robustly [40,48].

Several limitations are associated with the model, implementation, and generated interactions. GPT-4o generated both the chatbot and artificial-user responses, which may have produced linguistically and behaviorally compatible interactions and reduced observable misunderstandings, ambiguity, resistance, or off-protocol behavior. Each transcript also represents a stochastic interaction drawn from the configured chatbot and artificial user prompts. We did not estimate run-to-run variance by regenerating sessions from the same personas. Future evaluations should therefore test different model simulations, with repeated generations from the same artificial users.

The rating design also limits the precision with which we can separate chatbot performance from rater differences. Each full session was rated by a clinical expert, and a subset of 18 dialogues was rated a second time to provide preliminary information on rating consistency. In this subset, the absolute agreement for the Q-BAS mean was moderate (ICC [2,1]=0.55, 95% CI 0.15-0.80). Several factors likely contributed to this result. The subset was small, which widened the CI. ICC (2,1) is a stringent absolute-agreement index that incorporates systematic differences in rating severity between raters into the reliability estimate [49,50]. Licensed psychotherapists rated sessions descriptively higher than trainees, suggesting that differences in rater background may also have contributed to lower agreement. Many Q-BAS components also require subjective clinical judgment regarding whether a delivered behavior is adequate, which leaves room for legitimate disagreement. This moderate agreement means that component-level Q-BAS estimates should be interpreted as approximate and that the variance attributed to transcript-level differences may partly reflect rater severity or interpretation. More structural findings, completion of all 7 phases, and stronger ratings for clearly procedural components depend less on fine rater calibration and are correspondingly more robust. A larger multirater design is needed to estimate interrater reliability more precisely and strengthen component-level conclusions.

Finally, the findings are bounded by the study’s LLM, language, intervention, and sample size. The evaluation used a single model (GPT-4o), German-language interactions, and a structured intervention (behavioral activation); therefore, transferring the framework to other settings should not be assumed to be straightforward. A different model or prompting strategy would require its prompts to be re-engineered and retested because the fidelity patterns and failure modes we observed were tied to this implementation. A different language would require translation and cultural adaptation of both the intervention and the artificial users, together with rechecking that safety behaviors, such as crisis referral, still trigger correctly. A different psychotherapeutic approach may not map onto a component-based fidelity scale, such as the Q-BAS, and could require a different evaluation instrument and interaction design. Behavioral activation was well-suited here precisely because its structured, protocol-driven format made complete sessions measurable with a standardized scale. The sample of 48 sessions was determined by practical constraints rather than a formal power analysis [51]. Therefore, exploratory subgroup analyses in this small sample should be interpreted descriptively. Overall, the sample supported the clinical fidelity assessment and helped identify refinement targets. However, future studies should be appropriately designed to test subgroup differences and examine how well the framework transfers across models, languages, and interventions.

### Conclusion

We evaluated a GPT-4o–powered behavioral activation chatbot across 48 artificial user sessions rated by clinical experts. The chatbot completed all intervention phases and received stronger ratings for structured procedural components than for those requiring clinical judgment, therapeutic rapport, and adaptive redirection. The findings identify concrete refinement targets before human testing, especially more systematic verification of activity plans, rewards, and user-related concerns.

Artificial user simulations, combined with expert fidelity ratings, can identify protocol-level weaknesses before human testing. In this study, this value was clearest for structured behavioral activation components, interaction breakdowns in skeptical or information-seeking sessions, and safety scenarios that required more targeted testing. The next step is a staged evaluation that tests whether the observed fidelity patterns remain stable across different simulation models, human role-play, supervised studies with human users, and targeted safety scenarios.

For developers of similar systems, the findings point to 4 practical prompt-level refinement patterns: granular task breakdown, template-based content, embedded clinical decision rules, and explicit redirection mechanisms. These patterns should be treated as refinement hypotheses for future testing, rather than validated design principles. Complete prompts for both the behavioral activation chatbot and artificial users are provided in Multimedia Appendix 1 to support replication and further evaluation.

## Supplementary material

10.2196/94781 Multimedia Appendix 1 Results, prompt-refinement hypotheses, chatbot prompt, artificial user persona, and variation expressions for the behavioral activation chatbot evaluation.

## Abbreviations

BH: Benjamini-Hochberg
ICC: intraclass correlation coefficient
LLM: large language model
PHQ-9: Patient Health Questionnaire-9
Q-BAS: Quality of Behavioral Activation Scale
